# Cost-effectiveness Analysis of Hospital Infection Control Response to an Epidemic Respiratory Virus Threat

**DOI:** 10.3201/eid1512.090902

**Published:** 2009-12

**Authors:** Yock Young Dan, Paul A. Tambyah, Joe Sim, Jeremy Lim, Li Yang Hsu, Wai Leng Chow, Dale A. Fisher, Yue Sie Wong, Khek Yu Ho

**Affiliations:** National University Health System, Singapore (Y.Y. Dan, P.A. Tambyah, J. Sim, L.Y. Hsu, D.A. Fisher, K.Y. Ho); Singapore General Hospital, Singapore (J. Lim, W.L. Chow, Y.S. Wong)

**Keywords:** Pandemic (H1N1) 2009, pandemic, cost-effectiveness analysis, hospital infection control, nosocomial infections, influenza, viruses, expedited, research

## Abstract

The outbreak of influenza A pandemic (H1N1) 2009 prompted many countries in Asia, previously strongly affected by severe acute respiratory syndrome (SARS), to respond with stringent measures, particularly in preventing outbreaks in hospitals. We studied actual direct costs and cost-effectiveness of different response measures from a hospital perspective in tertiary hospitals in Singapore by simulating outbreaks of SARS, pandemic (H1N1) 2009, and 1918 Spanish influenza. Protection measures targeting only infected patients yielded lowest incremental cost/death averted of $23,000 (US$) for pandemic (H1N1) 2009. Enforced protection in high-risk areas (Yellow Alert) and full protection throughout the hospital (Orange Alert) averted deaths but came at an incremental cost of up to $2.5 million/death averted. SARS and Spanish influenza favored more stringent measures. High case-fatality rates, virulence, and high proportion of atypical manifestations impacted cost-effectiveness the most. A calibrated approach in accordance with viral characteristics and community risks may help refine responses to future epidemics.

## Abstract 2

Pandemic (H1N1) 2009 can be contained with less expensive measures than some other viruses.

Pandemic influenza A (H1N1) 2009 virus is a new influenza virus of swine origin that was first detected in April 2009. Within 4 months of its appearance in Mexico, it had spread to >100 countries, with >200,000 confirmed cases globally, including >2,000 deaths (*1*). When the World Health Organization (WHO) raised its global influenza pandemic alert to phase 5 (imminent pandemic) on April 27, 2009, many countries followed suit and activated their pandemic preparedness plans, although this varied between countries. Many countries with direct experience of the 2003 severe acute respiratory syndrome (SARS) outbreak tended toward more stringent measures.

Singapore was one of the countries most affected by SARS and experienced a disproportionate impact of the spread of the disease in hospitals (*2*,*3*). A total of 98 healthcare workers in Singapore were infected with SARS, 6 of whom died (*4*). After the SARS experience, Singapore’s Ministry of Health (MOH) developed a pandemic influenza plan with several levels of response that correlated roughly with the WHO Pandemic Alert Response system (*5*). The Disease Outbreak Response System (DORSCON)-FLU system that MOH devised requires progressively higher levels of infection control in hospitals in addition to border screening, restrictions on visitors to hospitals, and community-based syndromic surveillance for acute febrile illnesses (Table 1).

**Table 1:** Characteristics of Singapore MOH influenza outbreak response system*

Singapore MOH DORSCON alert level	WHO pandemic alert level	Global/local situation	Hospital measures	Community measures
Green 0	1	No novel influenza virus circulating	Triage and isolation of febrile patients, use of PPE as appropriate	Surveillance, maintenance of antiviral drug stockpile
Green 1	2–3	Novel virus but predominantly animal disease with limited transmission to humans	Full PPE for suspect cases, contact tracing for confirmed cases, antiviral treatment for all confirmed cases	Enhanced surveillance, communication, readiness measures
Yellow	4	Inefficient human-to-human transmission of novel virus	Full PPE for HCWs in high risk contact, visitor restriction, restrict movement of patients and HCWs	Enhanced surveillance, public health education, border body temperature screening, surveillance of returned travelers from affected areas
Orange	5	Global or local clusters but transmission still localized	PPE stepped up to cover “medium-risk” patients, no visitors, no interhospital movement of patients or HCWs, post-exposure prophylaxis for contacts	Body temperature screening at community areas, consider school closure, body temperature screening at borders, enhanced public health education
Red	6	Pandemic under way, import into Singapore is inevitable	As above with establishment of 18 influenza clinics	As above with possible use of masks in the community

*MOH, Ministry of Health; DORSCON, Disease Outbreak Response System; WHO, World Health Organization; PPE, personal protective equipment; HCWs, healthcare workers. Adapted from (*5*).

In accordance with the progressive elevation of WHO pandemic alert levels, Singapore raised its own pandemic alert level to Yellow on April 27, 2009, and further elevated it to Orange 2 days later. At this level, all hospital staff were required to wear N95 masks when dealing with all patients. Patients were restricted to 1 registered and screened visitor, all medical and nursing student rotations and local medical conferences were cancelled, leave restrictions for healthcare workers (HCWs) were put in place, interhospital movement of patients and HCWs was banned, and further limitations were placed on elective surgery. These measures were aimed primarily at avoiding a repeat of the SARS epidemic where nosocomial transmission originated with patients whose SARS infections were undiagnosed in hospital, and because influenza may be contagious before symptoms develop in infected patients. In fact, nosocomial influenza has been well documented since the 1957 Asian influenza pandemic (*6*). Based on studies conducted primarily in the United States, it has been estimated that 1 nosocomial case of influenza in a pediatric unit can cost up to $7,500 (US) (*7*). A recent review (*8*) of 28 nosocomial outbreaks of seasonal influenza summarized the evidence for nosocomial transmission of influenza in hospitals with accompanying illness and death (*8*,*9*).

When it subsequently became apparent that the case-fatality rate for pandemic (H1N1) 2009 was much lower than previously thought, especially in settings of industrialized countries, the alert level in Singapore was lowered to Yellow on May 11, 2009, even as WHO moved to alert level 6 after the pandemic was declared.

The risks and impacts of an outbreak will no doubt depend on the transmissibility, virulence, and clinical severity of illness. Thus, the benefits of a high alert status response at the onset of an outbreak as a “safe rather than sorry” strategy is not unreasonable when faced with an unknown novel potentially lethal virus. Yet, on the other hand, preventive measures from a hospital perspective come with a price. Direct costs include activation as well as ongoing administrative, manpower, and logistic resources, such as use of enhanced personal protective equipment, as part of the alert response measures.

We made use of this unique opportunity to evaluate the real costs of our primary prevention interventions and their potential cost-effectiveness against different models of influenza virulence and transmissibility in a simulated outbreak in our 1,000-bed tertiary teaching hospital to understand the relative incremental cost per additional death averted at different alert status levels. The key variables that affected the cost-effectiveness ratio the most were identified and studied. The same analysis was subsequently repeated for a parallel 1,500-bed tertiary teaching hospital. Using the outcome variables of disease cases, deaths, and incremental cost per death averted, we sought to determine if a calibrated and measured response plan based on characteristics of the virus in the outbreak could be better defined.

## Methods

To determine the cost incurred per day over the period where hospitals were at DORSCON Yellow and Orange, we obtained actual direct and indirect costs from the Operations and Finance Departments of the hospitals. Excess costs were measured by comparing these with operating costs and results over the same period in 2008.

**Figure 1:**
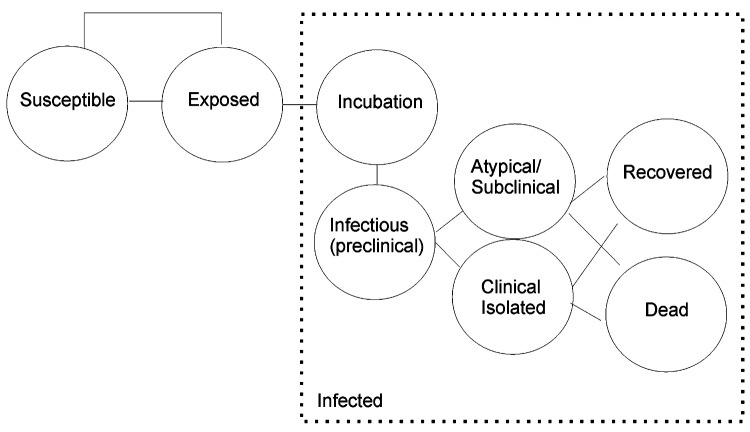
Markov model simulating a stochastic simulation of epidemics approach for an outbreak in a hospital institution.

To simulate a hospital outbreak, we used a decision analysis model to perform cost-effectiveness analysis to determine the impact of an outbreak from a single index case that was not detected by hospital surveillance and was found in the general ward. The Markov decision model was built using Treeage software (www.treeage.com), and simulation was performed based on hospital staff and inpatients (n = 7,500) over a time horizon of 30 days. Each person would transit between exclusive Markov states of Susceptible, Exposed, Incubation, Infectious, Isolated, Atypical, Recovered, or Dead. (Figure 1). The scenario assumes that all clinical cases will be identified and isolated. Infection is thus transmitted in the preclinical infectious phase or by atypical or subclinical cases not recognized and hence not isolated, as well as through the failure of personal protective equipment. Variables studied in the model included the number of persons exposed per infected patient, secondary attack rate, percentage of atypical and subclinical cases, duration of preclinical infectious period, and infectious period, as well as case-fatality rate.

Based on preliminary data available at the time of writing, comparisons were made between 3 respiratory viruses: a SARS-like virus, a 1918 Spanish influenza–like virus, and a pandemic (H1N1) 2009–type virus. Validation of the model was performed by comparing generated reproduction numbers to reported estimates from actual SARS data in Singapore (*10*) and Spanish influenza (for confined areas) (*11*) and showed consistent case and death numbers. We compared 4 different strategies: 1) no additional measures; 2) Green Alert response, which mandated personal protective equipment (PPE) for HCWs in direct contact with patients suspected of having avian influenza or other emerging infectious diseases; 3) Yellow Alert response, which mandated enhanced PPE at all high-risk areas; and 4) Orange Alert response, which mandated N95 masks for all patient contact and the restrictions described above (Table 1). Outcome measures were number of patients infected, number of deaths, cost (in US$) per case prevented, and cost per death prevented compared to baseline where no preventive measures were implemented, as well as incremental cost per death averted compared with the corresponding lower level of alert status. Multivariate sensitivity analysis was performed to understand the impact of viral characteristics as well as different hospital response policies on cost-effectiveness outcomes. The number of persons exposed in hospital and protective gear failure rates were from an actual outbreak simulation exercise performed at our hospital (*12*). Because there were no cases of pandemic (H1N1) 2009 virus infections during this period, estimates were all based on those in the literature (*13*). Details of the input variables are included in Table 2.

**Table 2:** Variables used in Markov mode (base case and sensitivity analysis) to compare outbreak estimates, Singapore*

Variable	Description	Base case	Sensitivity analysis
Exposure	No. persons exposed in 1 day in hospital per index case (nonlinear)	15 (average for 2 days) 6 (average for 5 days)	2–30
Secondary attack rate	No. persons exposed/infected	30% Spanish influenza 10% SARS 30% pandemic (H1N1) 2009	10–100%
Incubation period	Time to symptoms	Spanish influenza: 2 days SARS: 4 day Pandemic (H1N1) 2009: 3 days	1–7
Infectious period preclinical	Incubation–latent	Spanish influenza: 1 day SARS: 0 day Pandemic (H1N1) 2009: 1 day	1–3
% Clinical versus asymptomatic		Spanish influenza: 95% SARS: 100% Pandemic (H1N1) 2009: 95%	70–100%
% Atypical (missed)		Spanish influenza: 5% SARS: 20% Pandemic (H1N1) 2009: 5%	0–50%
% Complication		10× mortality rate	
Infective atypical	Infective	4 days	1–7
Case-fatality rate	% death	Spanish influenza: 5% SARS: 10% Pandemic (H1N1) 2009: 4%	
Isolation failure	Transmission despite PPE/isolation	5%	0­–10%
Exposure reduction	% reduction in exposure rate	Alert Green 50% Alert Yellow 80% Alert Orange 90%	0–100%
Cost based on alert policy, direct and indirect	Once Daily recurring	Activation: US$110,000 Green: US$4,000 Yellow: US$76,000 Orange: US$100,000	
Cost by type of treatment, based on actual financial charges	Isolation Treatment antiviral/day Uncomplicated influenza Complicated influenza Respiratory failure with mechanical ventilation	US$230 US$25 Mean: US$600, Median: US$420 Mean: US$1800, Median: US$220 Mean: US$5,500, Median:US$4,660	

*SARS, severe acute respiratory syndrome; PPE, personal protective equipment.

## Results

An outbreak of pandemic (H1N1) 2009 from introduction by an HCW, a patient with undiagnosed infection, or a visitor in our hospital at base case with no protection measures will result in 2,580 infected patients at 30 days. This finding would be similar to that of seasonal influenza and correspond to a 30% attack rate. With a 0.4% mortality rate, there would be 10 deaths from infection with pandemic (H1N1) 2009 virus. In contrast, Spanish influenza would result in 3,210 infected patients and 161 deaths (case-fatality rate 5%). The increased number of infections in the Spanish influenza model is driven by the short incubation time of the epidemic and results in more rounds of infection rather than an increase in basic reproductive number (average number of secondary cases per index case) (*14*). On the other hand, because SARS has a longer incubation period and lower transmissibility rate, the number of infected patients is lower at 825 but, owing to the high case-fatality rates, 82 deaths may ensue (Table 3).

**Table 3:** Results of cost-effectiveness analysis of potential outbreaks and responses, Singapore*

Alert level and disease	No. infected	No. deaths	Additional cost	Cost/case prevented†	Cost/death prevented†	Incremental cost/case‡	Incremental cost/death‡
None							
Pandemic (H1N1) 2009	2,580	10	25,200				
Spanish influenza	3,210	161	80,000				
SARS	825	83	99,200				
Green							
Pandemic (H1N1) 2009	316	1	326,430	95	23,644		
Spanish influenza	624	31	468,000	107	2,140		
SARS	105	11	220,500	120	1,195		
Yellow							
Pandemic (H1N1) 2009	59	0.2	1,485,500	414	103,274	3,221	827,907
Spanish influenza	120	6	2,212,000	493	9,857	2,472	49,829
SARS	43	4	1,188,000	995	9,945	11,146	121,241
Orange							
Pandemic (H1N1) 2009	24	0.1	1,836,000	506	126,807	7,153	2,503,600
Spanish influenza	59	2.95	2,856,000	629	12,590	7,541	153,333
SARS	12	1.2	1,537,000	1,263	12,601	8,041	7,541

*SARS, severe acute respiratory syndrome. All costs given in US$. †Compared with no policy. ‡Compared with 1 alert level down.

**Figure 2:**
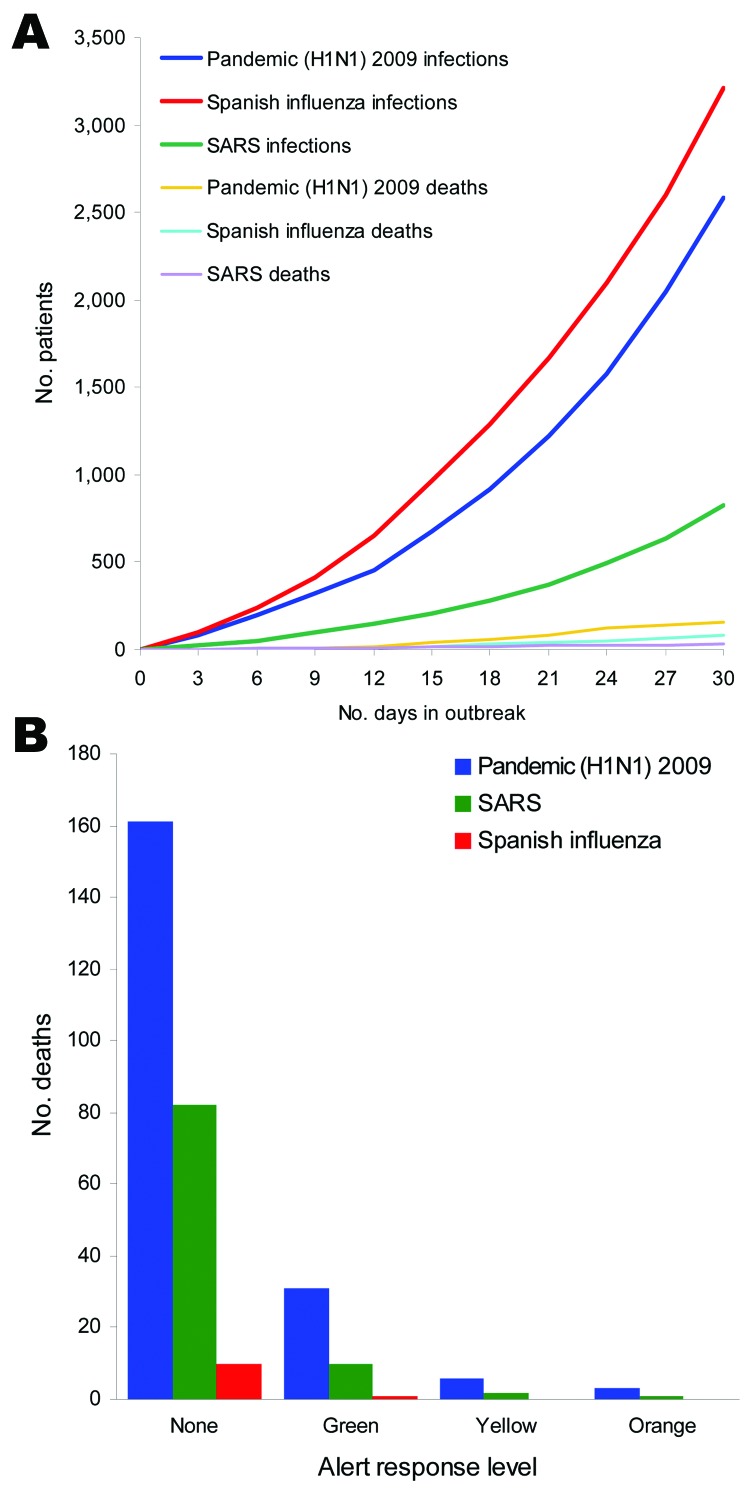
Epidemic simulation. A) Base case simulation assuming no protection over 30 days (n = 7,500). B) Number of deaths for pandemic (H1N1) 2009, Spanish influenza, and severe acute respiratory syndrome (SARS) with different levels of alert status.

**Figure 3:**
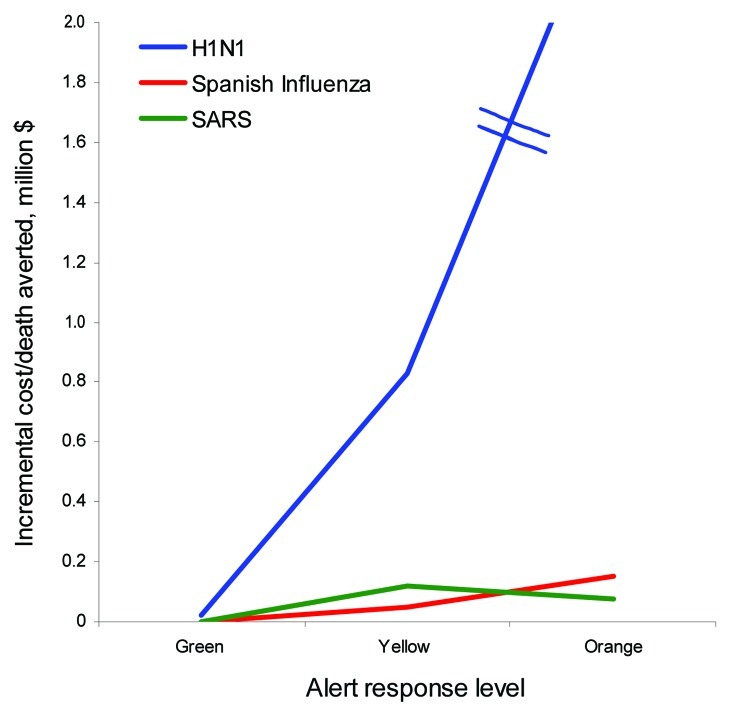
Incremental cost/death for 3 viruses with different alert status. Incremental cost to avert 1 additional death moving through ascending levels of alert status. Cost-effectiveness increases exponentially for pandemic (H1N1) 2009 while maintaining an almost linear fashion for both Spanish influenza and severe acute respiratory syndrome (SARS). The incremental cost/death averted ratio is lower for Alert Orange compared to Alert Yellow for SARS.

Green Alert status mandates PPE for HCWs in direct contact with patients suspected of having the infection. Transmission will thus be only through preclinical cases before they are identified and patients can be isolated or through atypical or subclinical cases that are missed. We assumed pandemic (H1N1) 2009 has a lowered 50% transmissibility for atypical or subclinical cases (*15*); this rate effectively reduced the infected patients to 316 with only 1 death (Figure 2, panel B). This resulted in additional costs of $95 to prevent 1 additional infected patient and $23,600 to prevent 1 death. Moving to Yellow Alert would reduce infected patients to 59 and avert all deaths. The costs to prevent additional infection and death are $3,221 and $828,000, respectively. Activating Orange alert with full PPE gear, restricting visitors, and cancelling elective procedures would halve the infections to only 24 cases with no deaths. However, the additional cost over Yellow Alert would escalate to $7,153 per infection prevented and a staggering $2.5 million to infinity for 1 death averted (Figure 3).

Simulation for Spanish influenza showed a decreased number of deaths from 31 at Green Alert to 6 at Yellow Alert and 3 at Orange Alert (Figure 2, panel B). This finding translated to $50,000 per death averted moving from Green to Yellow and $153,000 per death averted moving from Yellow Alert to Orange. For SARS, on the other hand, the incremental cost of moving from Green Alert to Yellow is $120,000 per death averted; this drops to $75,000 when moving from Yellow to Orange. This finding is mainly due to the high (10%) case-fatality rate and the relatively higher percentage of atypical patients who are missed and not isolated, a lesson learned from the actual SARS experience (Figure 3).

**Figure 4:**
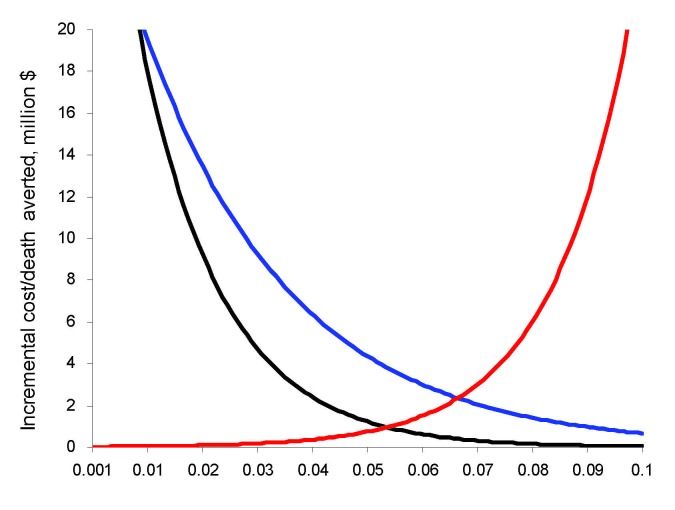
Sensitivity analysis for case-fatality rate (black line), % exposure reduction (red line), and secondary attack rate (blue line). Exponential graphs show poor cost-effectiveness at extremes of low case-fatality rate and low transmissibility (high % exposure reduction and low secondary attack rate).

Sensitivity analysis showed that the factors that impacted the cost-effectiveness ratio most are case-fatality rate, patient exposure rate, and secondary attack rate (Figure 4). In the pandemic (H1N1) 2009 scenario, the case fatality-rate ranging from 0.1% (seasonal influenza) to 10% (SARS) results in the cost per death averted moving from infinity (no death) to $35,000 per death averted (Orange Alert). Similarly, changing the exposure rate from 1.5 persons/day (10% PPE failure rate, Orange Alert) to 30 persons/day (0% reduction) per infected patient changed the incremental cost-effectiveness ratio from $2.5 million per death averted to $23,000. If pandemic (H1N1) 2009 had a higher 50% transmission rate, Orange Alert would become the most cost-effective strategy. The other variables had an impact on cost per case prevented but did not impact the incremental cost per death averted ratio.

To determine the impact of hospital size on our model, we modeled our simulation on the nation’s other tertiary hospital with 1,500 beds using their actual cost records. The model estimates that 10 expected deaths in the outbreak would be reduced to 1 death under Green Alert and none in Yellow and Orange Alerts. The incremental cost/death averted is $32,000, $1.9 million, and $5.4 million when moving from Green to Yellow to Orange, respectively. Although the cost ranking is consistent with that predicted by base-case simulation, the actual incremental cost index is much higher, reflecting the higher cost for activating alert status in a bigger hospital.

## Discussion

Singapore and many of the other countries badly affected by the SARS epidemic of 2003 launched comprehensive pandemic response plans based on a SARS model (*5*). The lessons of the SARS epidemic, in particular the effect of protecting HCWs from patients with undiagnosed, unisolated respiratory viral infections (*16*,*17*), have been applied rigorously to the pandemic plans of the Singapore Ministry of Health. Although it is difficult to quantify the impact of these interventions when they are taken as a whole, data from our modeling show that a nuanced approach that concentrates on administrative measures to isolate patients and selectively use PPE when working with patients suspected of having novel strains of pandemic (H1N1) 2009 virus would have a relatively favorable cost-effectiveness ratio.

On the other hand, the psychological and economic impact of SARS has been described as one of Singapore’s most traumatic experiences and one that left deep scars on the healthcare system of this country (*18*). It could be and has been argued that a draconian approach that seeks to protect all HCWs fully ensures that every case of influenza is identified early and contacts traced. Any healthcare facility ensuring no second- or third-generation transmission would provide intangible gains that exceed the economic costs of such a strategy. Nevertheless, this desire must still be balanced against the community impact of a disease such as influenza, which has a different epidemiology than SARS (*7*). We currently believe that pandemic (H1N1) 2009 virus causes predominantly community-based disease (*13*). Data from the United States, where infection control recommendations (*19*) are similar to our DORSCON Green, have not shown any evidence to date of large nosocomial outbreaks.

In our model, we have shown that cost-effectiveness ratio is dependent on the interplay between exposure rate, transmissibility (secondary attack rate), case-fatality rate, and risk of transmission from atypical cases. Infectious diseases with high fatality rates and transmission from atypical cases (such as SARS) will need the full benefit of PPE to reduce mortality rates. This finding is reflected in Orange Alert having a better cost-effectiveness ratio than Yellow Alert. Mild diseases with low fatality rates, such as pandemic (H1N1) 2009, and low incidence of atypical or subclinical infectious cases have the best cost-effectiveness ratio at Green Alert provided surveillance measures are able to identify infected patients and isolate them early. The cost-effectiveness ratio increases exponentially after that due to the much higher costs incurred. However, although Yellow Alert comes at a heavier price tag, it effectively averts any deaths. Activating Orange Alert increases cost with minimal benefit in mortality rate reduction. In reality, our model suggests that parallel efforts in contact tracing and voluntary quarantine may further reduce the exposure rate and break the chain of transmission.

Our base model took into account only direct costs associated with each alert status. In real situations, indirect costs such as lost revenues from cancellation of elective surgeries to free up hospital resources, decreased elective admissions and outpatient attendances, administrative costs associated with senior staff meetings, and lost clinical teaching time, add up to more than the direct costs and would magnify the incremental cost-effectiveness ratio further. In fact, if direct and indirect costs were included in the modeling, the incremental cost/death averted ratio of moving from Yellow Alert to Orange in pandemic (H1N1) 2009 increased to a staggering $8–$81 million for both hospitals. Although these indirect costs are not part of the infection control process per se, surge capacity response plans to ensure that the healthcare system has the reserve capacity to react to a full-blown community outbreak are critical to all pandemic plans (*20*) and contribute serious costs to the hospital.

The major limitations of our study are that we have simulated a situation in which community infection is still relatively low and the outbreak in hospital arises from 1 index case. When a community epidemic is established, the incidence of new index cases entering the institution increases, especially if there are prevalent atypical or subclinically infected persons. In such a scenario the cost-effectiveness ratio of higher alert status will decrease, and it may become more beneficial to escalate protective measures.

Cost-effectiveness analyses merely provide a mathematical projection to better understand the key factors that affect outcomes. The actual magnitude of the cost-effectiveness will vary depending on institutional cost, which varies between different sized hospitals and whether direct or indirect costs are included. Nonetheless, knowledge of the exponential relationship of the different viruses on the cost-effectiveness ranking is critical in charting response policy. Indirect costs of an uncontrolled pandemic are also economic and social, especially in Singapore where the economy is dependent on trade and tourism. A higher cost-effectiveness ratio does not imply that additional lives are not worth saving. In the case of pandemic (H1N1) 2009, if it costs $2.5 million to prevent 1 death, using a median age of 37 years for persons who died (*21*) and expected life expectancy of 80 years (*22*), the incremental cost-effectiveness ratio works out to $40,000 per life-year saved. In addition, preventive measures go beyond saving lives and include resultant savings from reducing hospitalization of infected patients and prolonged intensive care with mechanical ventilation for severe cases, as well as the logistic costs of further contact tracing and quarantine.

We have not factored the cost of influenza antiviral prophylaxis or the costs and effectiveness of novel vaccinations that may be required, nor did we include the costs of work-days lost from staff taking medical leave due to their being infected or being placed in quarantine. The impact of lax border controls, subclinical patients carrying the virus into the community, and closure of community institutions or even hospitals due to an outbreak were also not computed. We assumed that the hospital is a closed community with a fixed number of staff and patients. This obviously is not true in real life but is mitigated in our analysis because the same assumption is applied to every response measure and the outcomes are incremental indices over another level of protection.

From the perspective of a healthcare institution, how do we predict the virulence of new virus early in the outbreak and adopt the most cost-effective response policy? If a mild epidemic spreads rapidly through the community, there might be multiple points of entry into the hospital; however, such a mild community outbreak might present more commonly to primary healthcare clinics and presentations to hospital may be few. Thus a step-up approach from Green to Yellow in accordance with predicted risks as we have shown may be the most cost-effective approach.

It is not known for certain how pandemic (H1N1) 2009 will behave in subsequent waves. Although the new virus seems to have relatively low virulence, the virus might reemerge with a case-fatality rate more like that of the 1918 influenza pandemic or the SARS pandemic. Our model shows that DORSCON Green, which focuses on infection control for suspected cases, will achieve a relatively high degree of protection for our staff, patients, and visitors even in the setting of a higher case-fatality rate. The main advantage of DORSCON Yellow and Orange is that undetected infected persons that are not isolated are less likely to become a source of transmission if there is universal use of N95 masks. This has to be balanced with the degree of compliance that can be achieved by the use of full-scale PPE for patients with no risk of the disease (e.g., patients with trauma or other medical or surgical conditions) and the well known adverse effects of prolonged use of N95 masks (*23*).

However, it is useful to also note that although a step-up approach may be the most cost-effective for the healthcare institution, the appropriate policy stance at the national level may not necessarily be the same. Our model did not take into account the psychological and economic impact to the country and the larger healthcare system, which are serious factors to consider when making a policy decision on the appropriate response across the healthcare system. Singapore, Hong Kong, and China were among the settings most severely affected by the SARS outbreak in 2003. In the initial face of an unknown virus with a perceived high mortality rate in Mexico, Singapore’s response to first err on the side of safety and make adjustments dynamically as the situation became clearer therefore would be reasonable when viewed from the larger perspective.

Such actions, however, were not without their own adverse effects in terms of cost and in overall patient care at the healthcare institution. We had the opportunity to perform a cost-effectiveness analysis using the actual costs incurred from this heightened infection control response. We have quantified how the virulence or case-fatality rate of a respiratory viral infection has a serious impact on the hospital infection control response. This impact occurs at 2 levels, first, the actual number of deaths and ill persons, and second, the direct and indirect costs on the hospital in terms of activation, logistics, and lost revenue. This impact is reflected in the subsequent responses of Singapore and other countries when the virulence of the novel influenza virus appeared to be much less than previously feared. Understanding the key factors that affect the cost-effectiveness ratio will enable us to make better informed decisions as we prepare to respond to future epidemics.
